# Dicephalus-Bicollis Monstrosity

**Published:** 1891-07

**Authors:** Fred. H. P. Edwards

**Affiliations:** Iowa City, Iowa


### DICEPHALUS-BICOLLIS MONSTROSITY.

By Fred. H. P. Edwards,
Iowa City, Iowa.

On Sunday, January 18, 1891, I was called to attend a small heifer, three years old, which had been in labor about four hours and which the owner had attempted to deliver, and was informed by owner that the animal had gone its full time.

I found the animal lying on its right side apparently dead, having exhausted itself in attempting to deliver its offspring. Vaginal examination disclosed both fore feet and one head well in passage; the passage was rhy, dry and swollen. The head presented was torn badly, the damage having been done during the attempts at extraction; with lubrication I succeeded in entering womb and discovered what at first I thought was another calf, but on further examination could not find any other forelegs and at once saw I had a monstrosity. I oiled the passage well and succeeded in securing good hold in poll of head, presented with hook, strapped each leg and began gentle traction, but making very little headway owing to the animal not helping at all, determined to use all the force we had; and, after a few good, steady pulls, succeeded in delivering the calf with the other head placed back on its withers.

The calf weighed eighty-six pounds, was dead and much bloated. Mother was lacerated some, but made a speedy recovery. Externally calf looked normal, except right front leg at knee resembled more a hock than anything else.

There were fourteen cervical vertebra, the seventh pair being joined one to another: from the seventh cervical to the seventh dorsal they were joined by their transverse processes; from there on by their bodies The lumbar, sacral and coccygeal vertebræ were normal and single. The sternum was double and very abnormally broad. Ribs were normal, both in number and size.

The muscular system seemed healthy and in neck was double. The organs of digestion were perfectly developed and were double from mouth to stomach; from these on were single. There were two œsophagi which followed the usual course in neck and thoracic cavity, passed through same foramen in diaphragm, and entered rumen about two inches from each other. The liver was large and heavy.

The respiratory tract was double in cervical portion, there being two distinct tracheæ which had no connection whatever with one another, but entered the corresponding lung by a single trunk on its own side. The lungs were small and sank in water.

The urinary apparatus was single and well developed.

The heart was large, and normal. There were two anterior aortas and one posterior aorta. Each neck and head was well supplied with blood. The venous system was as well developed as the arterial, there being four jugulars, as well as the accessory jugulars, which were four in number and well developed.

The nervous system was perfect, the two brains being normal in size; the spinal cords were double up to about the seventh dorsal, where they joined and continued as one. The organs of sense were perfectly developed. The genital organs were normal, one testicle being in the scrotum and the other in the abdominal cavity.

